# Ayahuasca enhances functional connectivity in the third visual pathway and mirror neuron networks: a crossover, multiple-dose functional MRI study

**DOI:** 10.1093/scan/nsag004

**Published:** 2026-01-31

**Authors:** Carla Soares, Gisela Lima, Marta Teixeira, Rebeca André, Patrícia Rijo, Célia Cabral, Miguel Castelo-Branco

**Affiliations:** Coimbra Institute for Biomedical Imaging and Translational Research (CIBIT), University of Coimbra, Coimbra 3000-548, Portugal; Institute of Nuclear Sciences Applied to Health (ICNAS), University of Coimbra, Coimbra 3000-548, Portugal; Faculty of Medicine (FMUC), University of Coimbra, Coimbra 3000-548, Portugal; Coimbra Institute for Biomedical Imaging and Translational Research (CIBIT), University of Coimbra, Coimbra 3000-548, Portugal; Institute of Nuclear Sciences Applied to Health (ICNAS), University of Coimbra, Coimbra 3000-548, Portugal; Faculty of Medicine (FMUC), University of Coimbra, Coimbra 3000-548, Portugal; Coimbra Institute for Biomedical Imaging and Translational Research (CIBIT), University of Coimbra, Coimbra 3000-548, Portugal; Institute of Nuclear Sciences Applied to Health (ICNAS), University of Coimbra, Coimbra 3000-548, Portugal; Research Center for Biosciences & Health Technologies (CBIOS), Lusófona University, Lisboa 1749-024, Portugal; iMed.ULisboa, Faculty of Pharmacy, University of Lisbon, Lisboa 1649-003, Portugal; Research Center for Biosciences & Health Technologies (CBIOS), Lusófona University, Lisboa 1749-024, Portugal; iMed.ULisboa, Faculty of Pharmacy, University of Lisbon, Lisboa 1649-003, Portugal; Coimbra Institute for Clinical and Biomedical Research (iCBR), Clinic Academic Center of Coimbra (CACC), Faculty of Medicine, University of Coimbra, Coimbra 3000-548, Portugal; Center for Innovative Biomedicine and Biotechnology (CIBB), University of Coimbra, Coimbra 3000-548, Portugal; Department of Life Sciences, Centre for Functional Ecology, University of Coimbra, Coimbra 3000-456, Portugal; Instituto de Histologia e Embriologia, Faculty of Medicine, University of Coimbra, Coimbra 3000-548, Portugal; Coimbra Institute for Biomedical Imaging and Translational Research (CIBIT), University of Coimbra, Coimbra 3000-548, Portugal; Institute of Nuclear Sciences Applied to Health (ICNAS), University of Coimbra, Coimbra 3000-548, Portugal; Faculty of Medicine (FMUC), University of Coimbra, Coimbra 3000-548, Portugal; Faculty of Psychology and Neuroscience, University of Maastricht, The Oxfordlaan 55, Maastricht, 6229 EV, The Netherlands

**Keywords:** ayahuasca, psychedelics, pharmacoimaging, third visual pathway, mirror neuron system, social cognition

## Abstract

Understanding the neural mechanisms underlying the impact of psychedelics on social perception and cognition may be instrumental to unravel their therapeutic potential. We conducted a pharmacoimaging study to examine ayahuasca’s effects on a key theory of mind region, at the core of the third visual pathway (TVP)—the posterior superior temporal sulcus (pSTS), which is involved in facial emotion recognition and social perception. Twelve healthy participants (mean age: 40 ± 6.6 years; four females) completed a crossover design with three conditions: 0.5 mg/kg *N*, *N*-dimethyltryptamine (DMT), 0.8 mg/kg DMT, and placebo, with 1–2 months washout intervals. Resting-state functional magnetic resonance imaging (fMRI) was used to assess pSTS functional and effective connectivity. The highest dose significantly increased right pSTS connectivity and directed modulation from visual (primary and extrastriate cortices) and mirror neuron regions (supplementary motor cortex; SMC). Subjectively, this enhanced social cognitive states, with a strong positive correlation between pSTS–SMC connectivity and perspective-taking experiences. Additionally, ayahuasca produced positive psychological effects, including improved perceived social relationships, at 1-week follow-up despite minimal acute effects. Our findings reveal a novel mechanism of action of psychedelics at early stages of social information processing, with enhanced integration of the TVP and mirror neuron systems. The pSTS emerged as a critical hub supported by top-down and bottom-up evidence, providing a basis for understanding ayahuasca’s prosocial therapeutic effects.

## Introduction

Social cognition encompasses cognitive mechanisms that enable individuals to perceive, interpret, and respond to socially relevant stimuli, playing a crucial role in mental health and adaptive behaviour (Beaudoin and Beauchamp 2020). Underlying these processes are interconnected cortical and subcortical brain regions that form neural networks that encompass different levels of processing social stimuli. These networks range from the third visual pathway mediating dynamic perceptual cue processing during social interactions (Pitcher and Ungerleider 2021), to sensorimotor systems enabling action simulation through mirror mechanisms (Rizzolatti and Sinigaglia 2016), to the limbic system facilitating social-emotional processing (Bernhardt and Singer 2012), and to the theory of mind (ToM) network supporting mental state attribution (Schurz et al. 2014). In this work, we focus on a region at the intersection between the third visual pathway and the ToM network, the posterior superior temporal sulcus (pSTS), and how different doses of ayahuasca, which acts on 5-HT2A serotonin receptors, modulate functional and effective connectivity of this region and social cognition.

Within this framework, ToM/mentalizing emerges as a core component of social cognitive functioning (Schurz et al. 2021). Conceptualized as the ability to attribute and reason about others’ mental states while distinguishing them from one’s own, ToM enables the prediction and engagement in social interactions through the representation of others’ beliefs, intentions, and emotions (Frith and Frith 2003). Perspective-taking represents a specific form of mentalizing through which individuals represent others’ mental states by adopting their perspective (Quesque et al. 2024).

Connectivity-based meta-analytic approaches do support a network-level organization of the ‘social brain’, with integrative hub regions ensuring integration between lower-level sensory networks with higher-level associative systems (such as ToM) (Alcalá-López et al. 2018). The pSTS within the temporoparietal junction (TPJ) assumes particular relevance receiving inputs from the third visual pathway and mirror neuron system while supporting connectivity within the ToM network (Rizzolatti and Craighero 2004, Patel et al. 2019).

Current evidence suggests that social cognitive dysfunction emerges from disrupted neural circuits underlying social processes, involving both altered activity and connectivity within key regions of the ‘social brain’ and, in neurodegenerative disorders, structural atrophy in these networks (Porcelli et al. 2019). Impairments in ToM and emotion recognition constitute a core feature across neuropsychiatric disorders, including autism spectrum disorders, schizophrenia, mood disorders, and neurodegenerative conditions (Cotter et al. 2018). These deficits, particularly in mentalizing and facial emotion recognition, manifest as decreased motivation for social interaction, altered social decision-making, and social withdrawal (Porcelli et al. 2019). Consequently, social cognitive dysfunction contributes significantly to functional disability, representing a promising transdiagnostic domain that offers potential targets for novel therapeutic interventions.

The serotonin 5-HT2A receptor system is densely expressed throughout cortical regions implicated in ToM, such as the TPJ and medial prefrontal cortex (Beliveau et al. 2017), and emerging evidence suggests its potential role in social cognitive processes (Canli and Lesch 2007, Crockett et al. 2010). Pharmacological agents targeting this receptor system may therefore offer pathways to address social cognitive deficits. In this context, recent evidence has positioned psychedelics—which primarily act through 5-HT2A receptor agonism—as promising therapeutic agents, with findings suggesting that enhanced social cognition may constitute a significant mechanism underlying their therapeutic efficacy in conditions such as depression, post-traumatic stress disorder (PTSD), and addiction (Vollenweider and Preller 2020, Schmid and Bershad 2024).

These compounds have been shown to decrease within-network connectivity across associative cortical networks while increasing connectivity between brain regions underlying sensory function and perception, which may provide a potential to support therapeutic outcomes (Kwan et al. 2022). Of particular relevance is their impact on the Default Mode Network (DMN), which substantially overlaps with the ToM network (Soares et al. 2023). These substances produce characteristic phenomenological states, including ‘ego dissolution’ and experiences of unity, manifesting as a dissolution of boundaries between self and environment. These effects may influence self–other distinction—a fundamental component of mentalizing abilities.

Neuroimaging research with LSD has demonstrated that ego-dissolution effects correlate with altered activity in the TPJ (Tagliazucchi et al. 2016), a key node in the mentalizing network. LSD administration modulates self- and other-initiated social interactions, accompanied by attenuated activity in brain regions mediating self-processing and social cognition (Preller et al. 2018). Together with psilocybin, these substances have been shown to enhance empathy while reducing recognition of negative emotions and associated neural responses in limbic regions, potentially decreasing social withdrawal behaviours (Rocha et al. 2019, Olami and Peled-Avron 2024). These findings suggest a pattern of psychedelic-induced modulation of social cognition and its neural substrates.

Ayahuasca combines *N*, *N*-dimethyltryptamine (DMT), a serotonergic psychedelic, with β-carboline monoamine oxidase inhibitors, and presents a distinct pharmacological profile (Dos Santos et al. 2016). Beyond its primary action through 5-HT2A receptor agonism, ayahuasca’s compounds modulate multiple neural systems, including glutamatergic, dopaminergic, and sigma-1 receptors (Rossi et al. 2022). Emerging clinical evidence indicates therapeutic potential for ayahuasca in treating depression (Palhano-Fontes et al. 2019), with neuroimaging studies reporting altered functional connectivity in the DMN and limbic structures—neural circuits with implications to social cognition networks (Riba et al. 2006, Palhano-Fontes et al. 2015).

Despite growing interest in the therapeutic applications of psychedelics, understanding of how these compounds might modulate social cognition networks remains limited. While prior studies have begun to characterize ayahuasca’s effects on brain network connectivity, specific investigations of its impact on neural systems supporting ToM are lacking. Furthermore, the relationship between network-level neurobiological changes and social cognitive alterations remains poorly understood, particularly regarding dose-dependence.

The present study aims to address these knowledge gaps by examining ayahuasca’s dose-dependent effects on connectivity within a key ToM subregion underlying social perception. Considering that ToM subregions exhibit distinct connectivity patterns (Alcalá-López et al. 2018), we focused specifically on the pSTS due to its central role in social cognition and perception, as well as previous evidence of emotion recognition improvements following neurofeedback targeting the pSTS in social cognitive disorders (Direito et al. 2019, 2021, Patel et al. 2019). Thus, we investigated how different doses of ayahuasca modulate functional and effective connectivity of the pSTS and how these connectivity changes correlate with subjective reports of social cognition. Based on previous behavioural evidence demonstrating ayahuasca effects on social cognition dimensions, we hypothesized that ayahuasca administration would modulate pSTS connectivity and its integration with neural networks supporting mentalizing processes.

From a translational perspective, elucidating the neural and behavioural mechanisms of mentalizing modulation through ayahuasca administration offers promising pathways for developing targeted interventions to improve social functioning in individuals affected by neuropsychiatric disorders, such as autism spectrum disorders.

## Materials and methods

### Study design

**Figure 1.**
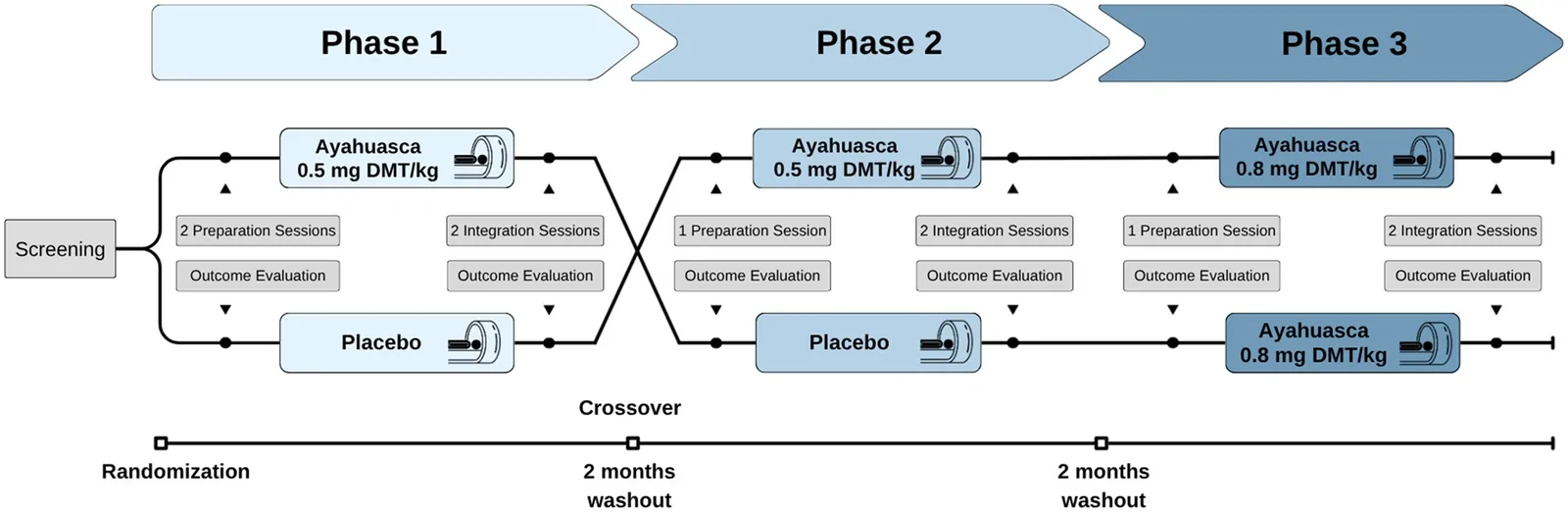
Study design. Participants (*N* = 12) were randomized to receive either the lowest dose of ayahuasca (0.5 mg/kg DMT) or placebo in the first phase, following a crossover design with a 1- to 2-month washout period between phases. Double-blinding was maintained through Phase 2, after which all participants received the highest dose of ayahuasca (0.8 mg/kg DMT) in a single-blind procedure during Phase 3. Each experimental session included pre-intervention remote preparation, baseline assessments, and post-intervention integration sessions and evaluations. Functional MRI data acquisition was conducted 40 min post-administration during each dosing session to capture peak pharmacodynamic effects.

We employed a crossover multiple dosage design, in which participants received ayahuasca with two concentrations of DMT (0.5 and 0.8 mg/kg DMT) and a placebo. The study followed a randomized, double-blind protocol for the lowest ayahuasca dose and placebo conditions, while the highest ayahuasca dose was administered last in a single-blinded manner for enhanced safety monitoring (Fig. 1).

The study was approved by the Ethics Committee of the Faculty of Medicine of the University of Coimbra and conducted in accordance with the principles of the Declaration of Helsinki. All participants provided written informed consent prior to participating in the study.

### Participants

The study included 12 healthy volunteers (4 female/8 male; mean age: 40 ± 6.6 years) with a minimum of 5 prior ayahuasca experiences. Exclusion criteria included psychiatric disorders, first-degree relatives with severe mental illness, major medical conditions, psychiatric, antihypertensive or sympathomimetic medication use, and pregnancy. The Mini International Neuropsychiatric Interview (MINI) was used to evaluate exclusion criteria (Lecrubier et al. 1997, Amorim 2000). Participants were required to abstain from psychoactive substances for 2 weeks pre-session and from caffeine, tobacco, and alcohol 24 h prior to administration.

Of 23 initially screened participants, 9 were excluded for not meeting eligibility criteria, 2 withdrew before study initiation, and 1 completed only two experimental conditions. The final sample comprised 12 participants (4 female; mean age: 40 ± 6.6 years), with prior ayahuasca experience. Table 1 presents detailed demographics, psychedelic use patterns, and baseline empathy traits.

**Table 1.** Sociodemographic characteristics, empathy traits, and patterns of psychedelic use among participants (*N* = 12).

Characteristics	*N* (%) or M (SD)
Age	40 (6.6)
Gender (F/M)	4 (33.3)/8 (66.7)
**Education**	
Upper secondary	1 (8.3)
Higher education	11 (91.7)
**Graffar socioeconomic index**	
Medium high	7 (58.3)
High	5 (41.7)
**Interpersonal Reactivity Index (IRI) scores (0–4)**	**M (SD)**
Perspective-taking	2.96 (0.19)
Fantasy	2.42 (0.21)
Empathic concern	2.97 (0.24)
Personal distress	1.12 (0.18)
**Psychedelic use lifetime ayahuasca use**	***N* (%)**
6–9	3 (27.3)
10–19	3 (27.3)
20–39	2 (18.2)
>40	3 (27.3)
**Last year ayahuasca use**	
0	3 (27.3)
1–5	5 (45.5)
6–39	2 (18.2)
>40	1 (9.1)
**Last month ayahuasca use**	
0	4 (36.4)
1–2	2 (18.2)
3–5	3 (27.3)
20–39	2 (18.2)
**Lifetime other psychedelics use**	
1–5	6 (54.5)
6–9	1 (9.1)
20–39	2 (18.2)
>40	2 (18.2)

### Procedure

The protocol included ayahuasca administration with non-directive psychological support, following safety guidelines for psychedelic research (Johnson et al. 2008). Clinical support from two psychologists and a medical doctor was available throughout all stages.

Following enrolment, participants received detailed protocol information, including evidence-based documentation on ayahuasca’s effects, risks, and benefits, along with preparation recommendations. To familiarize participants with the MRI environment and minimize adverse effects during acquisition, we provided didactic videos and MRI sound files. Remote preparation sessions aimed to establish rapport and trust, identify significant life events that could influence the psychedelic experience, discuss interaction boundaries, intentions, and expectations for the dosing session, and provide psychoeducation and coping techniques.

Dosing sessions were conducted individually in a private, comfortable room adjacent to MRI facilities, arranged to support the psychedelic experience. Each session lasted ∼7 h (12:00–19:00), with substance administration occurring at 14:00. Prior to dosing, participants completed pre-test assessments, underwent medical examination, and had time to engage in their usual preparation procedures and relaxation exercises. Music was available except during fMRI acquisition.

Following administration, participants were encouraged to engage in the experience with clinical support available as needed. At 40 min post-administration (estimated onset of effects), participants underwent fMRI acquisition for ∼50 min, then returned to the comfortable room. Once acute effects subsided, participants debriefed their experience and completed post-test assessments.

Integration sessions were conducted 1 day and 1 week post-dosing sessions to evaluate the experience’s impact, assess, and provide support for managing potential adverse effects, and beneficial outcomes. Semi-structured interviews and post-test assessments were administered.

### Ayahuasca quantification and placebo composition

Ayahuasca was prepared with the species *Banisteriopsis caapi* (Spruce ex Griseb.) C.V.Morton and *Mimosa tenuiflora* (Willd.) Poir. The quantification of *N*, *N*-dimethyl-1H-indole-3-ethylamine (*N*, *N*-DMT) present in the samples was performed on high-performance liquid chromatography with diode array detector (HPLC-DAD). The separation was performed with a reverse-phase column (Eclipse XDB-C18, 5 μm, 4.6 × 250 mm). The mobile phase was composed by methanol (A) and 0.3% trifluoroacetic acid in water (B). The elution was carried out in gradient mode from 30% (0 min) to 55% A (10 min) and again to 30% A (10–15 min), adapted from what was described by Santos et al. (2017) and internally reported as method ‘COIMBRA 2_SHORT’. The time of analysis was 15 min, including the stabilization of the RP-18 column, and the temperature was set at 25°C. The flow rate was 1 ml/min, the injection volume was 5 µl, and the detection wavelength was 278 nm. A triplicate of the sample (at a concentration of 1 mg/ml) was performed, using methanol as the solvent. *N*, *N*-DMT reference standard was prepared in a concentration range of 0.05–0.01 mg/ml. Compound identification was based on the comparison of retention time and ultraviolet spectra overlay with authentic standards. The limit of detection (LOD) and limit of quantification (LOQ) were calculated based on the standard deviation error of the response and the slope. LOD and LOQ were calculated as 3.3 and 10 σ/S, respectively, where σ is the standard deviation error of the response and S is the slope of the calibration curve. The ayahuasca preparation contained 2.44 ± 0.003 mg/ml of *N*, *N*-DMT.

For the placebo, for a 200 ml solution, we used 1 g of zinc sulphate heptahydrate, 400 mg of citric acid 1-hydrate, and 7.5% of caramel, which were poured to roasted barley tea at room temperature with stirring until complete dissolution. A homogenous brownish solution was obtained. For the preparation of the roasted barley tea, 10 g of toasted barley grains were added to 250 ml of water with stirring at boiling temperature for 15 min.

### MRI data acquisition

The MRI scans were acquired using a 3 T imaging system (MAGNETOM Prisma, Siemens Medical Solutions) with a 64-channel head coil. We collected resting-state functional images using a 7-min sequence and a total of 210 volumes. Participants were instructed to close their eyes and surrender to the experience, with the aim of minimizing any interference with their psychedelic experience. Resting-state BOLD fMRI scans had the following parameters: Repetition Time (TR) = 2000 ms; Echo Time (TE) = 20 ms; flip angle = 82°; slices = 50; slice thickness = 2.5 mm; field of view = 195 × 195 mm; voxel size = 2.5 × 2.5 × 2.5 mm. Additionally, we acquired T1-weighted anatomical MRI data at a spatial resolution of 1 × 1 × 1 mm^3^, with a TR of 2530 ms, TE of 3.5 ms, and a flip angle of 7°.

### fMRI data preprocessing and analysis

Preprocessing was conducted using CONN (Whitfield-Gabrieli and Nieto-Castanon 2012) (RRID: SCR_009550, 20.b) and SPM (Penny et al. 2011) (RRID: SCR_007037, 12.7771) in MATLAB R2022b. Steps included realignment with susceptibility distortion correction, slice timing correction, outlier detection, segmentation with Montreal Neurological Institute (MNI) normalization, and spatial smoothing [8 mm full width at half maximum (FWHM) Gaussian kernel]. Outlier identification used ART tool (thresholds: 0.9 mm framewise displacement, 5 SD BOLD signal changes) (Power et al. 2014). Denoising incorporated CompCor for white matter/CSF signals, motion parameters with derivatives, outlier scans, session effects, and linear trends (Nieto-Castanon 2020). Data were bandpass filtered (0.008–0.09 Hz).

### Region of interest selection

The region of interest (ROI) for connectivity analysis was selected based on the hypothesis that ayahuasca modulates regions of the mentalizing network, particularly the pSTS. The ROI was defined using meta-analyses of task-based fMRI studies investigating ToM (Soares et al. 2023). The pSTS ROI was centred at MNI coordinates (54, −50, 14) in the right superior temporal sulcus region with a volume of 1560 mm^3^.

### Functional and effective connectivity analysis

To assess ayahuasca’s effects on pSTS functional connectivity, we performed seed-to-voxel analysis using the pSTS ROI as the seed region. First-level analysis estimated connectivity using Fisher-transformed bivariate correlation coefficients from a weighted General Linear Model (GLM). Group-level analyses employed a GLM comparing connectivity across conditions (placebo, 0.5 mg/kg DMT, and 0.8 mg/kg DMT), using the Ayahuasca > Placebo contrast. Results were thresholded at a cluster-forming *P* < .001 at the voxel level and *P*-FDR (false discovery rate) < .05 at the cluster level.

To examine directional modulation of the pSTS under ayahuasca, we performed Granger Causality Mapping (GCM) between the pSTS and regions showing altered functional connectivity. Random-effects GCM analysis was conducted across all conditions, with ayahuasca-induced changes assessed via a subtraction method and significance determined by *t*-tests against absence of effect. Analysis was implemented in BrainVoyager 22.4.0.5046 (Brain Innovation, Maastricht, The Netherlands).

### Assessment of social cognition and subjective experience

Social cognition was assessed using the condensed version of the Multifaceted Empathy Test (MET-core-2) (Dziobek et al. 2008, Foell et al. 2018) and Visual Analog Scales (VAS). The MET-core-2 comprises 40 images depicting persons in emotionally salient situations. It assesses cognitive empathy through mental state attribution (selecting the correct emotion from four alternatives) and emotional empathy by asking participants to rate the extent to which they empathize with the depicted persons. The MET-core-2 was administered before and ∼4 h post-administration.

Subjective social cognitive states experienced during fMRI scanning were assessed with VAS using four items: ‘I thought about others’, ‘I thought about significant persons in my life’, ‘I placed myself in another person’s shoes’, and ‘I had feelings of empathy’. The first three items were adapted from the Amsterdam Resting-State Questionnaire (Alexander Diaz et al. 2013). Given strong intercorrelations between these items (*r* = 0.75–0.91, all *P* < .01) and acceptable internal consistency (Cronbach’s α = .77), a composite social cognition score was calculated by averaging the four items. VAS were administered the following day during integration sessions, after a guided recall exercise designed to help participants re-experience the scanning period. Baseline trait empathy was characterized using the Interpersonal Reactivity Index (Davis 1983, Limpo et al. 2010).

Acute psychedelic effects were assessed using the Hallucinogen Rating Scale (HRS) completed ∼4 h post-administration (Strassman et al. 1994). The evaluation of persisting effects was conducted 1 week post-administration using the Persisting Effects Questionnaire (PEQ) (Griffiths et al. 2006).

Behavioural and self-report data were analysed using repeated measures ANOVA examining effects of condition, and when applicable, time and scale. For non-normal distributions and substantial outliers, the Jonckheere–Terpstra test was employed to test ordered differences. Relationships between neural and behavioural measures were assessed using Spearman correlations. Analyses were performed in SPSS v28.0.0.0 with a significance threshold of *P* < .05, Bonferroni-corrected. Effect sizes were reported as partial eta-squared (ηp^2^).

## Results

### Acute effects of ayahuasca on posterior superior temporal sulcus connectivity

#### Ayahuasca increases posterior superior temporal sulcus functional connectivity with V1/V2 and supplementary motor cortex

**Figure 2.**
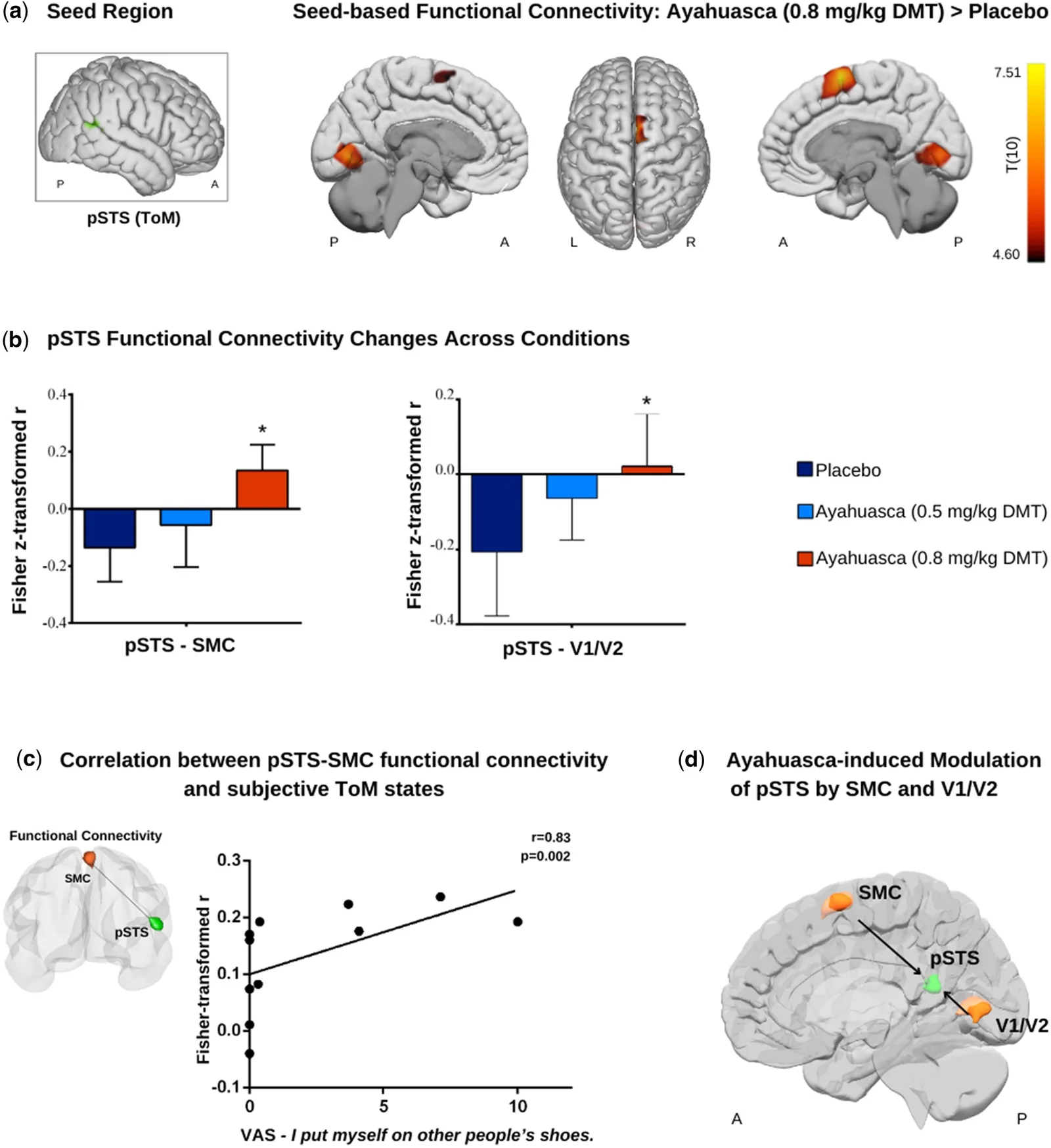
Ayahuasca modulation of pSTS connectivity. (a) Cortical maps show functional connectivity changes following ayahuasca administration (0.8 mg/kg DMT) compared to placebo (*P* < .05, cluster-size *P*-FDR corrected). (b) Graphs depict connectivity strength (Fisher-transformed correlation coefficients) for regions where pSTS showed altered functional connectivity across conditions. Asterisks indicate statistically significant changes between conditions. (c) Graph shows the Spearman correlation between the pSTS–SMC Fisher-transformed correlation values and the VAS item measuring subjective perspective-taking. (d) Illustration of Granger causality analysis revealing significant directional influences on pSTS from both SMC and V1/V2 at the highest dose of ayahuasca. pSTS, posterior superior temporal sulcus; SMC, supplementary motor cortex; V1/V2, primary and secondary visual cortex.

To investigate our hypothesis that ayahuasca modulates pSTS connectivity and its integration with other socially relevant neural systems, we conducted a seed-based connectivity analysis using the pSTS as the seed. Results revealed that the highest dose of ayahuasca increased functional connectivity between the right pSTS and two clusters (Fig. 2a): a visual cluster encompassing the bilateral intracalcarine cortex and lingual gyrus (*P*-FDR < .001, *k* = 559) and a second cluster involving the right supplementary motor cortex (rSMC) (*P*-FDR < .01, *k* = 298). Table 2 presents complete cluster information. Figure 2b shows an intriguing pattern of progression of functional connectivity across conditions, transitioning from an anticorrelation pattern during placebo and the lowest ayahuasca dose to positive correlations at the highest dose.

**Table 2.** Results of the seed-based functional connectivity analysis for the pSTS.

Peak cluster (*x*, *y*, *z*)	No. of voxels	Label	*P*-FDR corrected
+10, −76, +02	559	Intracalcarine cortex right Lingual gyrus right Lingual gyrus left Intracalcarine cortex left	.000484
+08, +02, +66	298	Supplementary motor cortex right Supplementary motor cortex left (two voxels)	.008549

Montreal Neurological Institute (MNI) coordinates represent peak locations of significantly increased connectivity with other brain regions. Results are shown at an FDR-corrected cluster-level threshold of *P* < .05.

To further understand the connectivity changes in the pSTS, we performed GCM to examine directional influences. Using GCM focused on regions showing significant functional connectivity changes, we found that the highest dose of ayahuasca revealed significant Granger-causal modulation of the right pSTS by both V1/V2 [*t*(11) = −2.80, *P* < .05] and the rSMC [*t*(11) = −3.33, *P* < .01] (Fig. 2d).

To test our hypothesis that altered pSTS functional connectivity would associate with subjective social cognition states during scanning, we calculated Spearman correlations between the Fisher-transformed correlation coefficients and the VAS measuring social cognition. A strong positive correlation emerged between the right pSTS–SMC functional connectivity and the subjective experience of perspective-taking (‘I put myself in other people’s shoes’) (*r*(11) = 0.83, *P* = .002, Bonferroni-corrected; Fig. 2c). No significant correlations were observed for other VAS items (all *P* > .05).

Collectively, these results suggest an ayahuasca-induced pattern of connectivity characterized by bidirectional modulation of the right pSTS: top-down from higher-order areas (rSMC) and bottom-up from early visual areas (V1/V2). This pattern suggests integration between the ToM network and the third visual pathway and mirror mechanism systems, facilitating reasoning about others’ perspectives.

Unmasked GCM analysis essentially replicated these findings (Fig. S1 and Table S1, see online supplementary material for a colour version of this figure and table), demonstrating altered effective connectivity within these social cognitive circuits.

#### Functional connectivity in control regions

To assess the specificity of the observed pSTS connectivity changes, we conducted a control analysis using the lateral prefrontal cortex (LPFC) as seed (left peak voxel: *x* = −43, *y* = 33, *z* = 28; right peak voxel: *x* = 41, *y* = 38, *z* = 30), a key hub of the frontoparietal network that is critical for cognitive control and working memory but not typically implicated in ayahuasca-induced effects (Santos et al. 2023). The seed-based connectivity analysis revealed no significant changes for either the left or right LPFC under any condition (*P* > .05, FDR-corrected). These null findings in regions associated with executive functions support the specificity of ayahuasca’s effects on the pSTS connectivity, suggesting that the observed modulation is not due to global changes in brain dynamics but rather represents a selective effect on specific regions.

### Effects of ayahuasca on behavioural and subjective measures

#### Reaction time differences across conditions on Multifaceted Empathy Test

**Figure 3.**
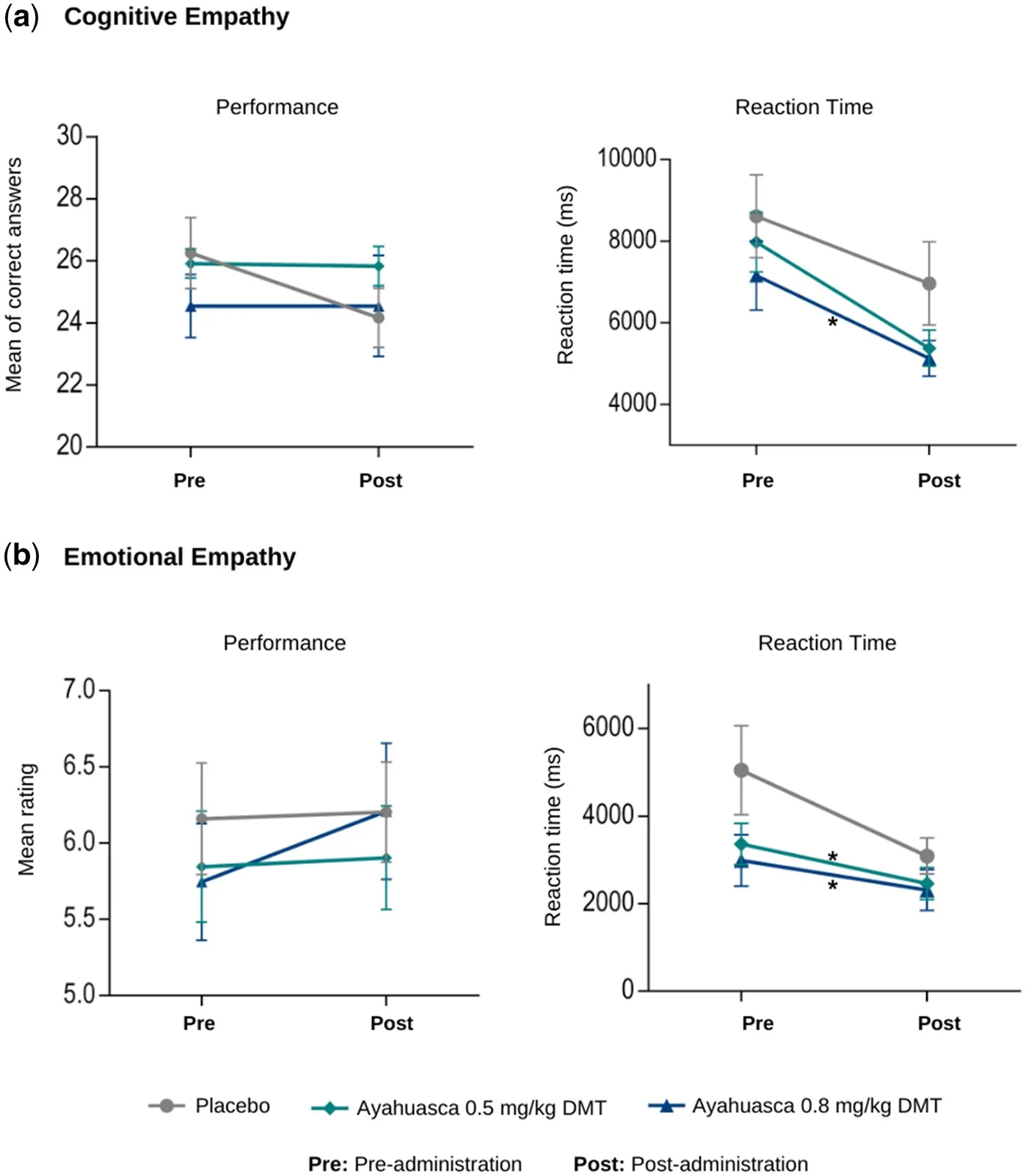
Ayahuasca effects on Multifaceted Empathy Test (MET). Performance and reaction times on cognitive (a) and emotional (b) empathy tasks across experimental conditions and time points. Cognitive empathy scores represent mean accuracy in mental state attribution from 40 scenarios. Emotional empathy scores correspond to mean empathy ratings toward depicted persons on a nine-point Likert scale. Error bars indicate standard error of the mean (SEM). Pre, before ayahuasca or placebo administration; Post, after administration. Asterisks represent statistically significant differences compared to placebo.

**Figure 4.**
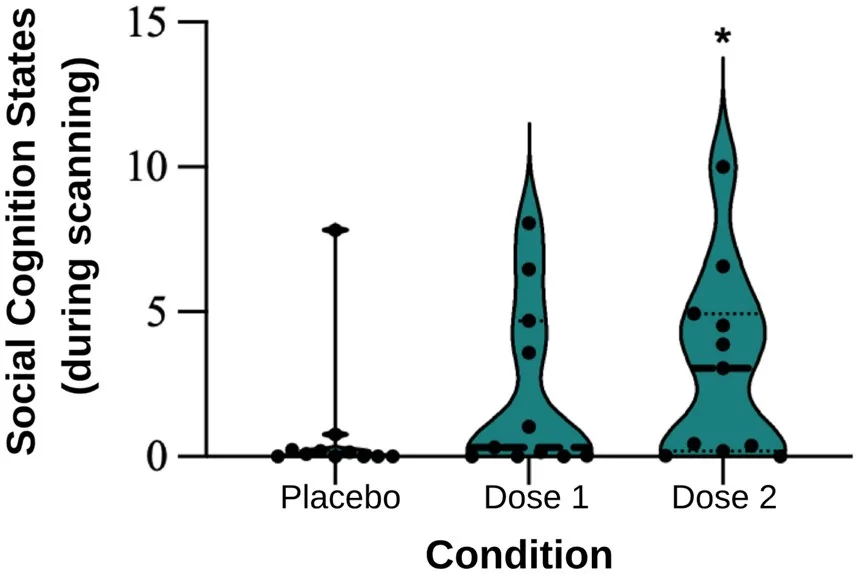
Subjective social cognition states during scanning. Visual Analogue Scales (VAS) measured subjective mentalizing and empathy experienced during scanning. Each VAS consisted of a 10-cm horizontal line, with endpoints denoting absence (0) and maximum intensity (10) of the effect. A significant ordered trend showed increasing social cognition states with higher ayahuasca doses. Dose 1: 0.5 mg DMT/kg; Dose 2: 0.8 mg DMT/kg. Individual values are represented by dots, while the median scores are depicted by lines. Asterisks represent statistically significant differences compared to placebo.

Repeated measures ANOVA for cognitive empathy reaction times revealed significant main effects of condition [*F*(2,20) = 3.52, *P* < .05, η_p_^2^ = 0.26] and time point [*F*(1,10) = 33.28, *P* < .001, η_p_^2^ = 0.77]. *Post-hoc* analyses with Bonferroni correction showed faster reaction times in the highest ayahuasca condition compared to placebo (mean difference = 1647 ms, *P* < .05) and in the post-administration time point compared to the baseline (mean difference = 2094 ms, *P* < .001). Similarly, for emotional empathy reaction times, significant main effects emerged for condition [*F*(2,20) = 12.32, *P* < .001, η_p_^2^ = 0.55] and time point [*F*(1,10) = 10.21, *P* < .05, η_p_^2^ = 0.51]. Pairwise comparisons with Bonferroni adjustment revealed faster responses in both the highest (mean difference = 1418 ms, *P* < .01) and lowest ayahuasca conditions (mean difference = 1160 ms, *P* < .05) compared to placebo, as well as at post-administration compared to baseline (mean difference = 1182, *P* < .05). Nevertheless, no significant condition × time point interaction was found for either measure (all *P* > .05). These findings are illustrated in Fig. 4. Means and standard deviations for all conditions are presented in Table S2 (see online supplementary material for a colour version of this table). Despite changes in reaction time, no significant differences were found for cognitive or emotional empathy scores (all *P* > .05; Fig. 3). The reduced reaction time in the ayahuasca condition could reflect faster processing of emotional stimuli and can also be attributed to task learning effects across conditions.

#### Enhancement of subjective social cognition states during the scanning

A Jonckheere–Terpstra test for ordered alternatives demonstrated a statistically significant trend of higher VAS social cognition median scores with higher dosages of ayahuasca (T_JT_ = 249.50, *z* = 2.26, *P* < .05), from placebo (Mdn = 0.10) to the lowest ayahuasca dose (Mdn = 0.31) to the highest ayahuasca dose (Mdn = 3.05) (Fig. 4). Pairwise comparisons with Bonferroni correction revealed a significant difference between the placebo and the highest ayahuasca dose (*P* < .05). These results suggest a dose-dependent relationship between ayahuasca administration and social cognitive experiences during resting-state, indicating ayahuasca’s potential in modulating social cognition.

#### Minimal effects of ayahuasca on Hallucinogen Rating Scale scores

A two-way repeated measures ANOVA (condition × dimension) revealed only a significant main effect for scale [*F*(1.42,14.18) = 13.96, *P* < .01, η_p_^2^ = 0.58], indicating differential sensitivity across HRS dimensions regardless of condition. Neither the main effect of condition [*F*(2,20) = 1.59, *P* > .05] nor the condition × dimension interaction [*F*(2.90,29.02) = 1.33, *P* > .05] reached significance. The absence of significant condition effects suggests minimal impact of the administered ayahuasca doses on overall subjective psychedelic experience. Additional exploratory analyses examining individual HRS dimensions, with visualization of HRS scores across experimental conditions, are provided in Fig. S2 (see online supplementary material for a colour version of this figure).

#### Ayahuasca induced positive subjective effects 1 week after administration

**Figure 5.**
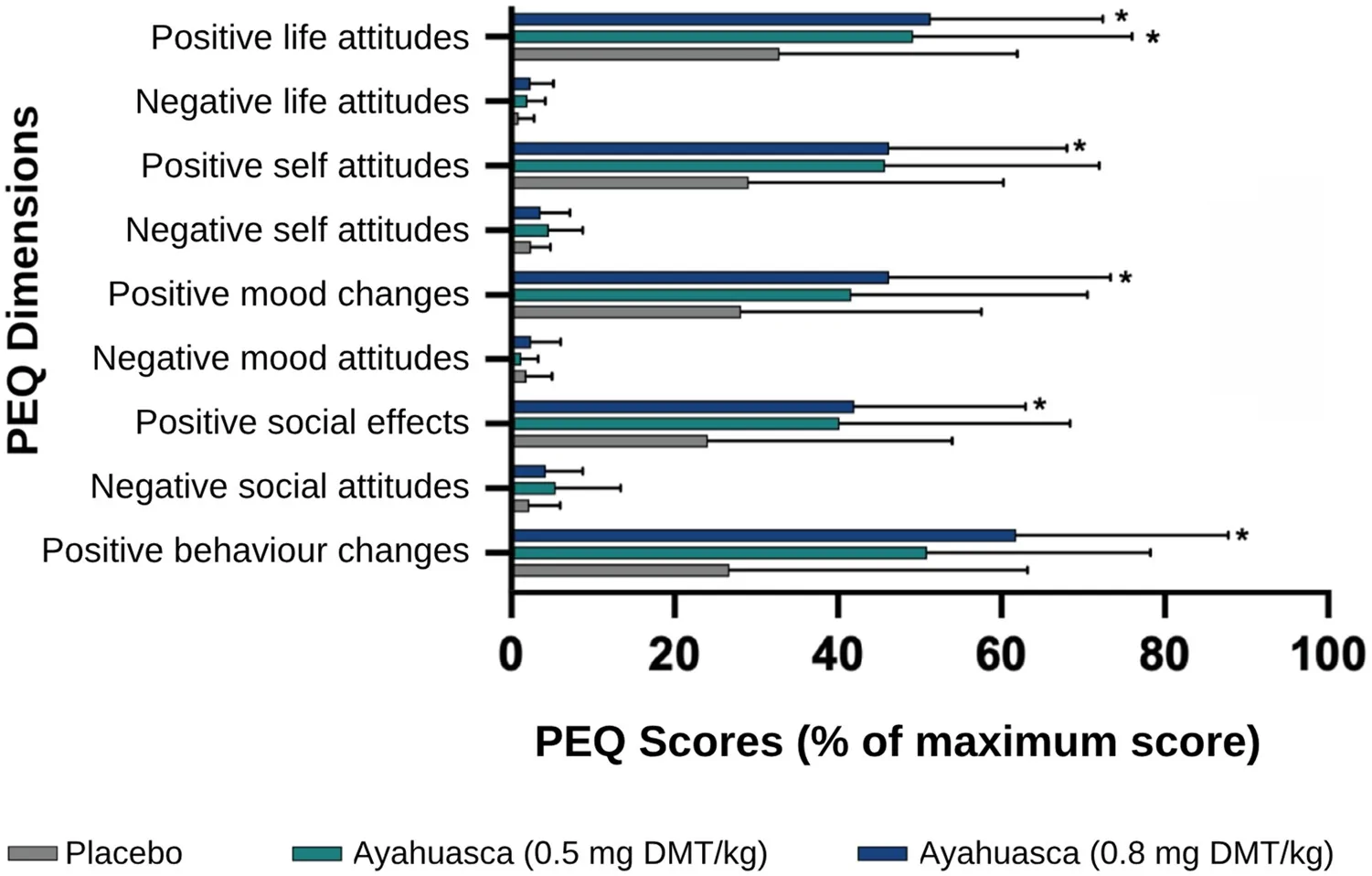
Persistent effects of ayahuasca at one week follow-up. Persisting Effects Questionnaire (PEQ) scores presented as mean percentage of maximum possible score for each dimension (*N* = 12). Error bars represent standard error of the mean (SEM). Asterisks indicate significant differences compared to placebo condition (*P < .05, Bonferroni-corrected).

Analysis of PEQ scores revealed a significant condition × dimension interaction [*F*(3.35,33.45) = 4.91, *P* < .01, η_p_^2^ = 0.33]. The main effects of condition [*F*(2,20) = 6.59, *P* < .01, η_p_^2^ = 0.40] and scale [*F*(1.23,12.29) = 26.17, *P* < .001, η_p_^2^ = 0.72] were also statistically significant. Consequently, simple main effects were further examined through one-way repeated measures ANOVAs for each scale across the three conditions. *Post-hoc* analyses showed that the highest ayahuasca dose, compared to placebo, increased scores on positive attitudes, mood changes, social effects, and behavioural changes (all *P* < .05, Bonferroni corrected). The lower dose only increased positive attitudes about life (*P* < .05, Bonferroni corrected). No significant differences emerged on negative dimensions (all *P* > .05). Figure 5 shows these results and Tables S3 and S4 (see online supplementary material for a colour version of these tables) present detailed statistics for the comparison of each scale across conditions.

## Discussion

This study investigated ayahuasca’s modulatory effects on a ToM subregion at the core of social perception and associated behavioural outcomes, motivated by evidence suggesting that social cognition pathways underlie the therapeutic efficacy of psychedelics. Our findings show that ayahuasca enhances directed modulation of the pSTS—a key node of the ToM network and the third visual pathway—by the primary and extrastriate visual cortices, as well as the SMC, which is part of the mirror neuron system. Notably, the strength of SMC–pSTS functional connectivity positively correlated with enhanced subjective perspective-taking scores during the scanning session. Collectively, these findings demonstrate that ayahuasca modulates processing within the ‘social brain’ by altering early stages of social cognition, involving the third visual pathway and mirror neuron mechanisms, with the pSTS emerging as a central hub integrating bottom-up and top-down processing.

The observed modulation of the pSTS by early visual areas in the highest ayahuasca condition implicates the third visual pathway, which projects from V1, via the motion-selective middle temporal area, to the pSTS and selectively responds to dynamic social cues such as facial expressions and body movements (Pitcher and Ungerleider 2021). Distinct from the ventral and dorsal pathways that process object identification and spatial relationships, respectively, the third visual pathway is specialized for social visual processing, supporting higher-order cognitive functions. Thus, it is essential for mentalizing abilities, with the pSTS as a critical node integrating visual and auditory information essential for processing others’ mental states and intentions (Küçük et al. 2024). Our findings suggest that ayahuasca increases the integration of internally generated visual information with higher-order social cognitive processes, potentially enhancing understanding of others’ perspectives.

Concurrently, the increased pSTS-directed modulation by the SMC, whose connectivity strength correlated with subjective perspective-taking, suggests a complementary pathway for social cognitive processing. The SMC (part of the mirror neuron system) plays a critical role in linking cognition to action (Nachev et al. 2008), serving as an interface between motor planning and higher-order cognitive processes. While associated with movement planning and motor imagery, the functions of SMC extend to internal simulation of actions, predominantly driven by internal rather than external cues. The rostral–caudal organization of the SMC, from pre-SMA’s cognitive processing to SMA’s motor execution (Nachev et al. 2008), positions it as a key structure for integrating cognitive and motor aspects of social action understanding. The observed positive correlation between pSTS–SMC connectivity and subjective perspective-taking scores suggests functional relevance for social cognition. The SMC brain activity has been associated with the mirror mechanism, which supports understanding others’ actions by simulating them through one’s own sensorimotor representations (Rizzolatti and Sinigaglia 2016). According to the embodied simulation proposal, we can have an immediate understanding of others’ mental states and emotions through motor resonance (Gallese 2007). Our findings suggest that the enhanced pSTS–SMC connectivity may facilitate automatic simulation processes and embodied mechanisms, contributing to the increased subjective perspective-taking reports under ayahuasca.

Our observation of enhanced pSTS connectivity with visual and supplementary motor pathways complements previous findings of reduced connectome idiosyncrasy after collective ayahuasca intake (Mallaroni et al. 2024), suggesting that these specific network reconfigurations may contribute to the increased similarity in functional connectivity patterns observed across individuals and interbrain synchrony, potentially facilitating shared neural representations that support social cognition.

These findings provide insights into existing neurocognitive models of psychedelic action, revealing mechanisms within social cognition networks that complement current conceptual frameworks. While the REBUS model (Carhart-Harris and Friston 2019) proposes that psychedelics broadly reduce the top-down constraining influence of high-order cortical networks on lower-level processing, our observation of enhanced SMC–pSTS connectivity appears to contrast with this framework. This suggests that within social cognition circuits, ayahuasca may instead specifically strengthen certain top-down connections from action planning to social-perceptual regions.

Regarding visual processing, the strong prior model (Corlett et al. 2019) predicts reduced bottom-up visual inputs under psychedelics, yet we observed enhanced connectivity from visual cortices to the pSTS. This finding aligns with the cortico-striatal thalamo-cortical model’s (Vollenweider and Preller 2020, Doss et al. 2022, Kwan et al. 2022) emphasis on sensory information amplification, though specifically within the third visual pathway. These results emphasize the value of examining psychedelic effects within specific functional networks, an approach that may be particularly important for understanding therapeutic mechanisms.

The enhanced subjective reports of social cognitive states in the highest ayahuasca dose align with previous research demonstrating that psychedelics modulate social cognition (Preller and Vollenweider 2019). Research has shown that LSD modulates social feedback processing through enhanced activity in the medial prefrontal cortex, with these effects mediated by 5-HT2A receptor activation and leading to increased adaptation to similar group opinions (Duerler et al. 2020). Our findings raise the hypothesis that this modulation may relate to psychedelic-induced changes in the early stages of social cognition, which could alter how individuals process others’ perspectives and social feedback, thereby potently increasing receptivity to interpersonal information in meaningful social interactions.

We observed faster responses without significant improvements in emotion recognition and empathy as measured with the Multifaceted Empathy Test (MET), which suggests improvement of emotional processing efficiency or learning effects due to the absence of an interaction effect between condition and time point. Meta-analytic evidence using this measure suggests that classical psychedelics typically enhance emotional empathy without affecting emotion recognition (Olami and Peled-Avron 2024). Our sample, constituted by healthy volunteers with prior ayahuasca experience and relatively high baseline empathic traits, may have created ceiling effects that limited our ability to detect significant changes. Nevertheless, our findings align with previous experimental studies reporting no significant effects of ayahuasca on empathy (Rossi et al. 2023) and recognition of facial emotional expressions (Rocha et al. 2021). Similarly, these studies only found differences in reaction times. It is noteworthy that previous studies with ayahuasca reporting increased empathy were primarily conducted in retreat settings with MET administration occurring the morning after ceremonial practices (Kiraga et al. 2021, Uthaug et al. 2021), which suggests that contextual factors may influence the effects of ayahuasca on empathy.

The divergence between the null findings on the MET scores and enhanced subjective social cognition states measured via VAS in our study suggests that low doses of ayahuasca may preferentially modulate mentalizing processes during introspective states. This pattern is consistent with the DMN alterations typically induced by psychedelics (Gattuso et al. 2023), a neural system critically involved in introspection and social cognition. Our findings add to these results, suggesting ayahuasca modulates the ongoing subjective experience during resting states favouring mentalizing states, even in the absence of pronounced psychedelic effects, acknowledging that functional connectivity changes could reflect distinct phenomenological experiences.

This study revealed a distinctive temporal profile of ayahuasca-induced subjective effects, characterized by minimal acute alterations but significant changes at 1-week follow-up. The minimal acute effects on the HRS indicate that both administered doses were below typical subjective effects thresholds. Our observations did not reproduce somaesthesia effects at 0.5 mg DMT/kg and affective, cognitive, and perceptual alterations at 0.7 mg DMT/kg (Riba et al. 2001), suggesting variability in ayahuasca’s subjective effects and potentially complex interactions between ayahuasca components.

Nevertheless, significant effects emerged at the 1-week follow-up assessment, particularly in the domains of positive attitudes, mood, social effects, and behavioural changes, predominantly in the highest dose condition. The selective influence of the lowest dose on positive attitudes suggests a dose-dependent relationship in ayahuasca’s post-acute effects. The absence of significant changes on negative scales indicates limited adverse outcomes in healthy participants under controlled conditions. Importantly, the dissociation between acute and persisting effects suggests that the post-acute changes may reflect distinct mechanisms such as sustained alterations in neural network connectivity or psychological integration processes. Further research on the relationship between ­connectivity changes and persistent behavioural effects would be valuable to understand the mechanisms underlying ayahuasca’s persisting effects.

While this study elucidates the effects of ayahuasca within social brain networks, several limitations warrant consideration. First, our sample consisted of experienced users, which may constrain the generalizability of the findings. Long-term ayahuasca use has been associated with structural cortical changes (Bouso et al. 2015). Although such alterations were not observed specifically in the pSTS, regular use may nonetheless influence baseline connectivity patterns or modulate the network’s functional response to acute ayahuasca administration. Second, directed connectivity was assessed using GCM, which identifies interactions based on the temporal precedence of observed BOLD signals (Roebroeck et al. 2005). While GCM is an effective analytic tool, its application to fMRI can be sensitive to regional variations in hemodynamic response function latency, temporal downsampling, and measurement noise (Friston 2009, Seth et al. 2013). With a TR of 2.0 s, the sampling rate is slower than the underlying neuronal dynamics, potentially limiting the detection of rapid reciprocal coupling. Consequently, our results reflect statistical precedence rather than millisecond-level neuronal causality. We nonetheless selected GCM for its exploratory capacity, as it remains agnostic to the *a priori* model specifications required by Dynamic Causal Modelling (DCM), an advantage for this novel investigation of ayahuasca’s impact on the social brain. Given that GCM and DCM can yield qualitatively consistent estimates in fMRI (Bajaj et al. 2016), our findings establish a directional framework that future hypothesis-driven studies can further refine using DCM.

## Conclusion

In summary, our study demonstrates that ayahuasca modulates social perception and high-level cognition networks while concurrently altering subjective social cognitive states. The enhancement of both visual and supplementary motor pathways’ directed connectivity with the pSTS may reflect a mechanism whereby increased integration of visual processing with mirror mechanisms supports embodied understanding of others’ perspectives.

## Supplementary Material

nsag004_Supplementary_Data

## Data Availability

Data will be made available upon reasonable request to the ­corresponding author.
