# Supplementary material for: Ayahuasca enhances functional connectivity in the third visual pathway and mirror neuron networks: a crossover, multiple-dose functional MRI study
Source: Soc Cogn Affect Neurosci. 2026 Jan 31;21(1):nsag004. doi: 10.1093/scan/nsag004 (PMC12936398; doi:10.1093/scan/nsag004)
Supplement: nsag004_Supplementary_Data [file nsag004_supplementary_data.docx]

**Supplementary Material**

**Figure S1. Whole-brain Granger Causality analysis for the pSTS seed region.** Cortical maps illustrate Granger Causality Mapping (GCM) changes in the highest ayahuasca dose (0.8 mg DMT/kg) compared to placebo (p<0.01, cluster corrected). Orange represents regions influencing the pSTS seed region (sources), and blue represents regions influenced by the seed (targets).

**
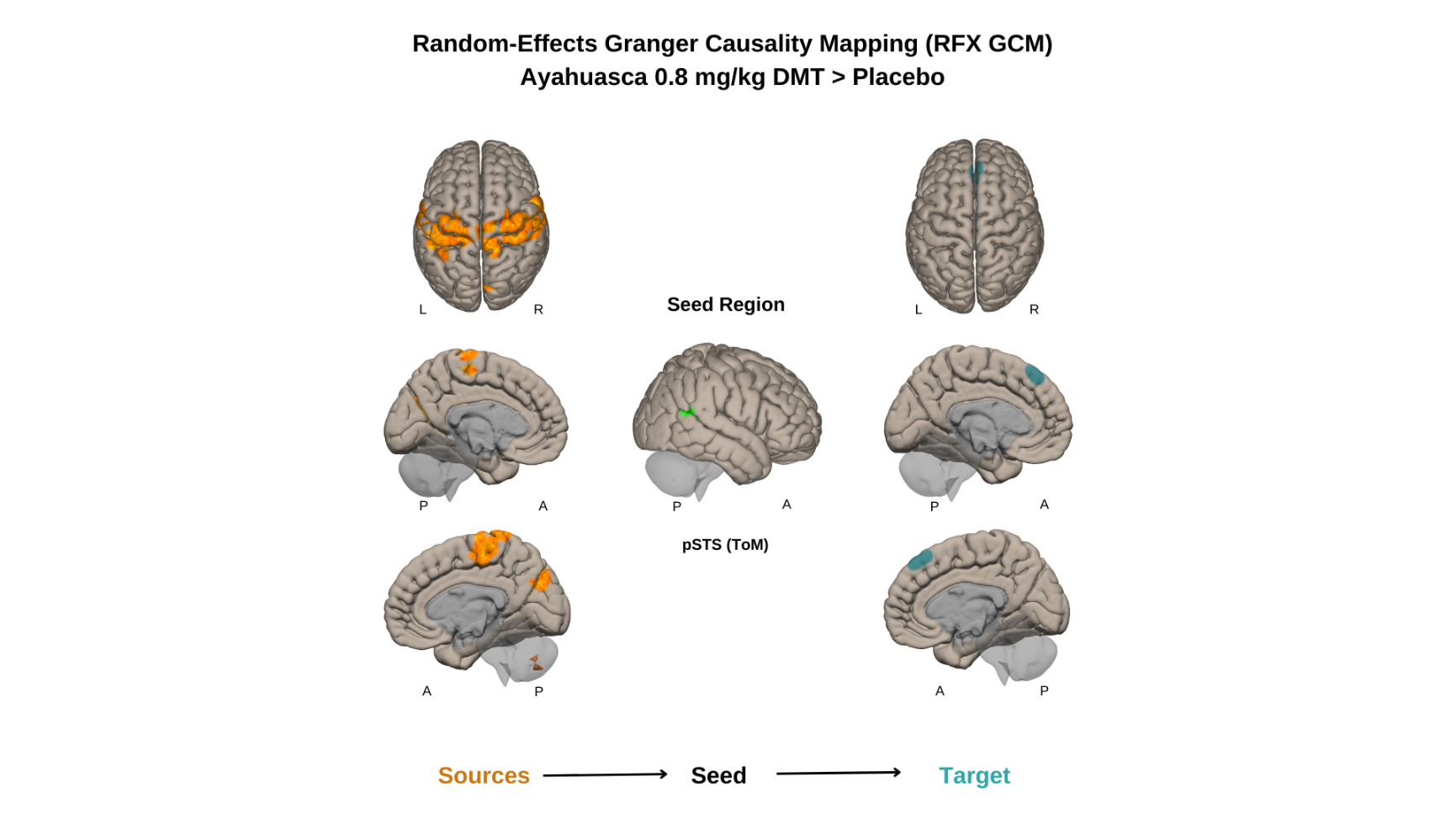
**

**Table S1. Results of whole-brain Granger Causality Mapping (GCM) analysis for the pSTS seed region.** Peak coordinates in MNI space, cluster sizes, direction of causality, and statistical values for regions showing significant Granger causality differences between the highest ayahuasca dose (0.8 mg DMT/kg) and placebo. Negative t-values indicate regions influencing the pSTS (sources), while positive t-values indicate regions influenced by the pSTS (targets).

| **Peak Cluster**  **(x, y, z)** | **Nr of**  **voxels** | **Label** | **H** | **Direction** | **t** | **p** |
| --- | --- | --- | --- | --- | --- | --- |
| +45, -4, +67 | 10170 | Precentral gyrus/Premotor cortex  Primary motor cortex  Postcentral gyrus/Primary somatosensory cortex | R | Source | -6.786242 | 0.000048 |
| +60, +8, +31 | 780 | Premotor cortex | R | Source | -5.577917 | 0.000235 |
| +33, -70, -50 | 803 | Cerebellum | R | Source | -4.521468 | 0.001106 |
| +21, -70, +28 | 1355 | Cuneous/Visual-associative cortex | R | Source | -5.840978 | 0.000164 |
| +9, -37, +73 | 2727 | Postcentral gyrus / Primary somatosensory cortex | R | Source | -5.526403 | 0.000252 |
| 0, +35, +52 | 974 | Medial prefrontal cortex | R/L | Target | 6.703768 | 0.000053 |
| -21, -64, +19 | 1415 | Retrosplenial cortex  Secondary visual cortex  Cuneous/Visual associative cortex  Precuneous | L | Source | -5.448765 | 0.000281 |
| -18, -31, +76 | 8413 | Precentral gyrus/Primary motor cortex | L | Source | -6.375042 | 0.000081 |
| -57, -1, +28 | 950 | Precentral gyrus/Premotor cortex | L | Source | -5.029768 | 0.000514 |

H: hemisphere; R: right; L: left

**Table S2. Means (M) and standard deviations (SD) for Multifaceted Empathy Scores (MET) across conditions and time points.**

| **Measure** | **Condition** | **Pre-administration**  **M (SD)** | **Post-administration**  **M (SD)** |  |
| --- | --- | --- | --- | --- |
| Cognitive Empathy  Reaction Time (ms) | Placebo | 8614.52 (3365.15) | 6965.52 (3385.70) |  |
|  | Lowest ayahuasca dose | 7977.70 (2421.34) | 5376.41 (1457.94) |  |
|  | Highest ayahuasca dose | 7159.55 (2797.66) | 5127.13 (1443.59) |  |
| Emotional Empathy  Reaction Time (ms) | Placebo | 5049.69 (3362.04) | 3089.28 (1374.47) |  |
|  | Lowest ayahuasca dose | 3363.61 (1565.99) | 2456.20 (1216.58) |  |
|  | Highest ayahuasca dose | 2990.62 (1941.95) | 2312.79 (1549.25) |  |
| Cognitive Empathy  (correct answers) | Placebo | 25.82 (3.87) | 23.73 (3.07) |  |
|  | Lowest ayahuasca dose | 25.73 (1.56) | 25.73 (2.28) |  |
|  | Highest ayahuasca dose | 24.55 (3.36) | 24.55 (5.41) |  |
| Emotional Empathy  (Mean rating) | Placebo | 6.09 (1.31) | 6.18 (1.19) |  |
|  | Lowest ayahuasca dose | 5.76 (1.28) | 5.89 (1.23) |  |
|  | Highest ayahuasca dose | 5.75 (1.27) | 6.21 (1.48) |  |

Note: The lowest dose corresponds to 0.5 mg/kg DMT and the highest dose is equivalent to 0.8 mg/kg DMT.

**Exploratory Analysis of Individual Hallucinogen Rating Scale (HRS) Dimensions**

Following our primary analysis, we conducted separate one-way repeated measures ANOVAs for each HRS dimension (Intensity, Somaesthesia, Affect, Perception, Cognition, and Volition) with condition (placebo, lowest dose, highest dose) as the only within-subject factor. When significant effects were found, post-hoc comparisons were conducted using Bonferroni correction for multiple comparisons.


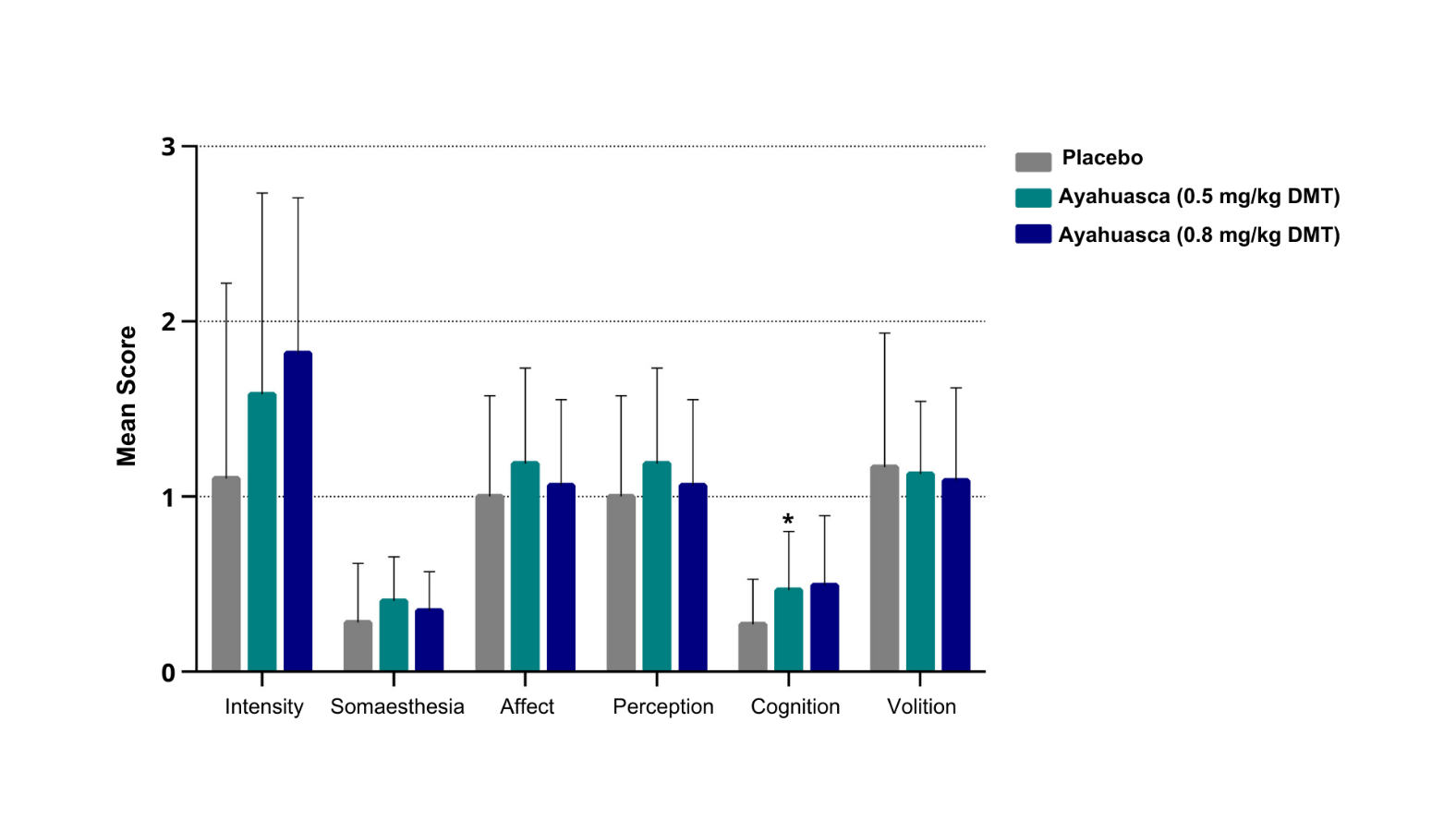
Among all HRS dimensions, only Cognition showed a significant main effect of condition (F_(2,20)_ = 4.33, p < 0.05, η_p_^2^=0.30). Post-hoc analyses revealed that the lowest dose condition (M=0.46, SD=0.35) showed significantly higher scores compared to placebo (M=0.29, SD=0.26; p<0.05). While the highest dose condition showed the highest mean scores (M=0.49, SD=0.40), the large individual variability limited statistical significance after Bonferroni correction (p>0.05). Figure S3 illustrates the results.

**Figure S2. HRS scores across conditions.** Bar chart showing mean HRS subscale scores and error bars representing the standard deviation following administration of placebo and the two doses of ayahuasca. The exploratory analysis indicated a significant difference in the Cognition subscale (*p<0.05).

**Analysis of** **Persisting Effects Questionnaire (PEQ) Across Conditions**

**Table S3. Persisting Effects Questionnaire (PEQ) Results: Comparison Across Conditions at One-Week Follow-up**

PEQ scores are presented as mean (M) percentages of maximum possible score with standard deviation (SD) (N=11). Additional questions are reported as mean raw scores (range: 0-8), except for the final item (range: -3 to +3). The Negative Behavioral Changes scale was excluded from subsequent analyses as it showed no variance across conditions, with all participants reporting zero changes regardless of dose.

| **PEQ Scales** | **Placebo**  **M(SD)** | **Low dose**  **M(SD)** | **High dose**  **M(SD)** |
| --- | --- | --- | --- |
| Positive life attitudes | 33.57 (30.44) | 49.23 (26.77) * | 51.33 (21.07) * |
| Negative life attitudes | 0.98 (1.98) | 1.96 (2.19) | 2.38 (2.78) |
| Positive self-attitudes | 30.74 (32.10) | 45.79 (26.19) | 46.28 (21.76) * |
| Negative self-attitudes | 2.31 (2.45) | 4.63 (4.10) | 3.64 (3.54) |
| Positive mood changes | 28.28 (30.83) | 41.62 (28.92) | 46.26 (27.12) * |
| Negative mood changes | 2.02 (3.21) | 1.21 (2.08) | 2.42 (3.65) |
| Positive social effects | 26.06 (30.55) | 40.20 (28.20) | 42.02 (20.93) * |
| Negative social effects | 2.22 (3.98) | 5.45 (7.91) | 4.24 (4.49) |
| Positive behavioral changes | 29.09 (37.27) | 50.91 (27.37) | 61.82 (26.01) * |
| Negative behavioral changes | 0.00 (0.00) | 0.00 (0.00) | 0.00 (0.00) |
| **Additional questions** |  |  |  |
| Personal Meaningfulness | 2.91 (1.58) | 3.55 (1.75) | 3.55 (1.13) |
| Spiritual Significance | 2.55 (1.63) | 3.09 (2.12) | 3.18 (1.40) |
| Psychological Challenge | 2.27 (1.74) | 2.45 (1.57) | 2.45 (1.29) |
| Personal Insight | 2.27 (1.79) | 3.36 (2.06) | 3.27 (0.79) |
| Well-being/Life Satisfaction Change | 1.27 (1.10) | 2.00 (1.10) | 1.82 (0.87) |

Note: Asterisks indicate significant difference from placebo condition (p<0.05).

**Table S4. Significant Changes in PEQ Scores Across Experimental Conditions at One-Week Follow-up.** Statistically significant results from repeated measures ANOVA assessing the effects of condition and scale on PEQ. For significant effects, F-statistics, p-values, and effect sizes (partial eta squared, ηp^2^) are presented along with Bonferroni-corrected pairwise comparisons (mean differences and 95% confidence intervals). Non-significant scales (p>0.05) are omitted.

| **PEQ Scale** | **F** | **p-value** | **η_p_^2^** | **Significant Pairwise Comparisons*** | **Mean Difference [95% CI]** |
| --- | --- | --- | --- | --- | --- |
| Positive life attitudes | F(2,20)=7.35 | <0.01 | 0.42 | Placebo < Lowest dose  Placebo < Highest dose | 15.66 [0.52, 30.81]  17.76 [0.99, 34.53] |
| Positive self-attitudes | F(2,20)=5.46 | <0.05 | 0.35 | Placebo < Highest dose | 15.54 [1.01, 30.07] |
| Positive mood changes | F(2,20)=5.93 | <0.01 | 0.37 | Placebo < Highest dose | 17.98 [5.12, 30.84] |
| Positive social effects | F(2,20)=5.52 | <0.05 | 0.36 | Placebo < Highest dose | 15.96 [0.99, 30.93] |
| Positive behavioral changes | F(2,20)=5.86 | <0.01 | 0.37 | Placebo < Highest dose | - 1. [3.49, 61.97] |

*****Multiple comparisons were corrected using the Bonferroni method to control for Type 1 error.
